# Palliative care as a shared language: Reflections from an ELNEC seminar in Salzburg

**DOI:** 10.1017/S1478951526102405

**Published:** 2026-04-20

**Authors:** Júlia Drummond de Camargo

**Affiliations:** Palliative Care Support Team, Hospital Samaritano Higienópolis, São Paulo, Brazil

Palliative care is often described through its conceptual lineage, most notably the work of Cicely Saunders, whose vision centered on the relief of suffering, the promotion of quality of life, and the provision of holistic, person-centered care for individuals facing life-threatening illness (Saunders 2001). Since the emergence of the modern hospice movement, palliative care has expanded globally. Yet this expansion has not always been accompanied by proportional investment in education and continuous professional development – particularly for nurses, who remain closest to patients throughout much of the illness trajectory.

As a nurse, I encountered end-of-life care early in my professional training. Those initial experiences shaped my understanding of what it means to accompany patients and families during moments of profound vulnerability. There is a form of fulfillment in this work that resists precise explanation: a quiet sense of purpose rooted in presence, continuity, and attentiveness. For many years, I assumed that this understanding was shared only within limited professional or cultural contexts. That assumption shifted when I encountered the End-of-Life Nursing Education Consortium (ELNEC) and recognized that this language of care extended far beyond my own setting.

## The ELNEC experience

The ELNEC is an international educational initiative founded and led by Betty R. Ferrell, with the mission of preparing nurses to deliver high-quality palliative and end-of-life care. Over more than 25 years, ELNEC has become a global reference in palliative nursing education, distinguished by its comprehensive curriculum, evidence-based foundations, and its “train-the-trainer” pedagogical model (Ferrell et al. 2005; Godzik, 2025).

A central partnership sustaining ELNEC has been its collaboration with the Open Medical Institute (OMI), whose mission is to provide intensive educational opportunities for health professionals from diverse regions through week-long seminars held in Salzburg, Austria. ELNEC remains the only nursing program invited to participate in this initiative and has been offered regularly since its first course in 2006 (Paice et al. 2025).

In August 2025, I received an unexpected invitation to attend the ELNEC International Seminar scheduled for January 2026 in Salzburg. My immediate response was disbelief, quickly followed by apprehension. I immersed myself in learning more about the program and its facilitators, while questioning how tangible this experience would be in practice. Another concern emerged almost immediately: my proficiency in English.

Brazil is internationally classified as a country with low English proficiency. In the 2024/2025 EF English Proficiency Index, Brazil ranked 81st among 116 countries, with a score below the global average (EF Education First 2024). Accepting an invitation to participate in an intensive academic seminar conducted entirely in English was intimidating. Declining, however, never felt like a real option. There was an intuitive certainty – difficult to articulate, yet impossible to ignore – that I needed to be there.

## A multicultural space of learning

Arriving in Salzburg was both disorienting and inspiring. Stepping off the train into subzero temperatures, I became acutely aware that I was the first Brazilian nurse to participate in an ELNEC seminar. What neither the facilitators nor I could have anticipated was the significance this presence would assume.

The seminar brought together 34 nursing fellows from 14 countries and was facilitated by 6 experienced nurse leaders from the United States and Europe. Over 5 consecutive days, the program combined lectures and workshops, practical laboratories focused on pain management, symptom control, and communication, a leadership workshop, and country-based project presentations. The curriculum followed the core ELNEC modules, including pain and symptom management, ethical and legal issues, communication, cultural and spiritual care, loss and grief, and care in the final hours and days of life (Godzik, 2025).

The learning environment was intentionally structured to be welcoming and psychologically safe. Despite wide differences in health systems, legislation, and cultural norms, a shared understanding emerged quickly. The language of palliative care proved stronger than linguistic barriers.

## Communication, vulnerability, and shared humanity

The modules addressing communication and care in the final hours of life were particularly impactful. These sessions created space for vulnerability, emotional expression, and collective reflection. Participants shared personal stories, ethical tensions, and clinical uncertainties within an environment marked by mutual respect and empathy. Such moments echoed what the literature consistently affirms: communication in serious illness care is not intuitive – it is a clinical competency that requires deliberate training, reflection, and practice (Back et al. 2007; VitalTalk 2019).

In palliative care, language often unfolds within uncertainty rather than resolution. Communication does not merely transmit information; it helps clinicians and patients inhabit time differently, sustaining presence when prognosis remains indeterminate (Geber-Junior and Forte 2025a). Increasingly, communication is understood not as adherence to scripts or protocols but as a relational process that precedes content and depends on trust, emotional attunement, and shared meaning (Geber-Junior and Forte 2025b). Engaging in these conversations within a multicultural group made visible a central truth: while cultural expressions may differ, the experience of suffering associated with serious illness is universal.

These shared narratives reinforced the importance of interdisciplinary alignment, peer support, and self-care as essential components of sustainable palliative care practice.

## What this experience changed

Beyond the formal curriculum, informal interactions strengthened connections among fellows and fostered the emergence of an international community of practice. The ELNEC train-the-trainer model reinforced a sense of responsibility – not only to acquire knowledge but to translate and adapt it within local contexts upon returning home.

The seminar concluded with the “Blessing of the Hands,” led by Betty R. Ferrell: a symbolic ritual honoring the hands through which nurses deliver care, comfort, and compassion. This moment crystallized much of what the week had represented. It reaffirmed my commitment to nursing, to palliative care, and to myself – not only as a professional but as a person.

## Reflections for palliative practice

This experience underscored that palliative care is not merely a clinical specialty but a shared language of care that transcends borders, cultures, and professional identities. Participation in the ELNEC International Seminar proved transformative in ways that extended beyond technical learning. It fostered professional confidence, personal growth, and a renewed sense of belonging.

The experience also reinforces the ELNEC model as a powerful strategy for strengthening palliative care education globally, highlighting the central roles of education, leadership, and self-care. In a field where compassion fatigue and burnout are well documented – particularly among professionals engaged in end-of-life care – such integrative educational spaces are not optional; they are essential for sustaining the workforce (Duarte and Pinto-Gouveia 2017). Importantly, emerging empirical evidence supports this perspective, demonstrating that limited training in palliative care and ethics is associated with more paternalistic, obstinate, or consumerist decision-making patterns, whereas structured education correlates with greater respect for patient autonomy and shared decision-making at the end of life (de Camargo and Forte 2024).

What I ultimately brought back from Salzburg was not only knowledge but a deeper sense of belonging: to palliative care, to nursing, and to myself.
